# Aflatoxin B1 Detection Using a Highly-Sensitive Molecularly-Imprinted Electrochemical Sensor Based on an Electropolymerized Metal Organic Framework

**DOI:** 10.3390/toxins7093540

**Published:** 2015-09-07

**Authors:** Mengjuan Jiang, Mohamed Braiek, Anca Florea, Amani Chrouda, Carole Farre, Anne Bonhomme, Francois Bessueille, Francis Vocanson, Aidong Zhang, Nicole Jaffrezic-Renault

**Affiliations:** 1 University of Lyon, Institute of Analytical Sciences, UMR-CNRS 5280, La Doua Street, 5, Villeurbanne 69100, France; E-Mails: mohamed_braiek@yahoo.fr (M.B.); florea.ancas@yahoo.com (A.F.); amani.chrouda@yahoo.fr (A.C.); carole.farre@univ-lyon1.fr (C.F.); anne.bonhomme@isa-lyon.fr (A.B.); francois.bessueille@univ-lyon1.fr (F.B.); 2 Key Laboratory of Pesticide and Chemical Biology of Ministry of Education, College of Chemistry, Central China Normal University, Wuhan 430079, China; E-Mails: mjjiang@mails.ccnu.edu.cn (M.J.); adzhang@mail.ccnu.edu.cn (A.Z.); 3 University of Lyon, Laboratoire Hubert Curien, UMR 5516, Jean-Monnet University of Saint-Etienne, Saint-Etienne F-42023, France; E-Mail: francis.vocanson@univ-st-etienne.fr

**Keywords:** electrochemical sensors, aflatoxin B1, molecularly imprinted polymers, metal organic framework, gold nanoparticles

## Abstract

A sensitive electrochemical molecularly-imprinted sensor was developed for the detection of aflatoxin B1 (AFB1), by electropolymerization of *p*-aminothiophenol-functionalized gold nanoparticles in the presence of AFB1 as a template molecule. The extraction of the template leads to the formation of cavities that are able to specifically recognize and bind AFB1 through π-π interactions between AFB1 molecules and aniline moities. The performance of the developed sensor for the detection of AFB1 was investigated by linear sweep voltammetry using a hexacyanoferrate/hexacyanoferrite solution as a redox probe, the electron transfer rate increasing when the concentration of AFB1 increases, due to a p-doping effect. The molecularly-imprinted sensor exhibits a broad linear range, between 3.2 fM and 3.2 µM, and a quantification limit of 3 fM. Compared to the non-imprinted sensor, the imprinting factor was found to be 10. Selectivity studies were also performed towards the binding of other aflatoxins and ochratoxin A, proving good selectivity.

## 1. Introduction

**Figure 1:**
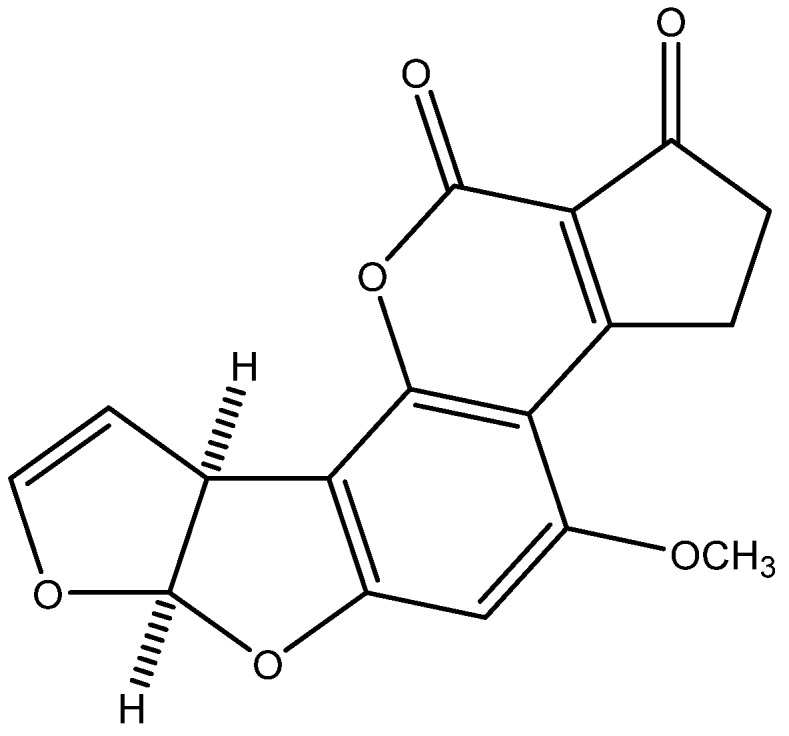
Chemical structure of aflatoxin B_1_.

Aflatoxin B1 (Figure 1) (CAS No. 1162-65-8) whose formal name is 2,3,6a*R*,9a*S*-tetrahydro-4-methoxy-1*H*,11*H*-cyclopenta[c]furo[3',2':4,5]furo[2,3-h][1]benzopyran-1,11-dione is an aflatoxin produced by *Aspergillus flavus* and *A. parasiticus*. It is arguably the most potent carcinogen known, and is up to twice as carcinogenic as an equitoxic dose of X-rays. Aflatoxin B1 can permeate through the skin. Dermal exposure to this aflatoxin, in particular environmental conditions, can lead to major health risks. AFB1 has a strong toxicity to the human and some animals and it is the main cause of liver damage. The minimum admissible level of AFB1 in food products should be 20 µg/kg [1,2].

Therefore, determining aflatoxin B1 mainly in almonds, Brazil nuts, hazelnuts and pistachios, and dried figs and other food is very important. Several methods have been reported for the quantification of aflatoxin B1 in food, such as thin layer chromatography (TLC) [3], liquid chromatography (LC) [4], enzyme linked immunosorbent assay (ELISA) [5], liquid chromatography-tandem mass spectrometry (LC-MSMS) [6]. However, these methods suffer from being time consuming, require expensive instrumentation, complex pretreatment of samples, and skilled staff. Increasing efforts have been focused on using electrochemical sensors to measure the concentration of mycotoxins because of their short response time, convenience, high sensitivity, and low cost.

The molecular imprinting technique has been extensively applied in preparation of polymer materials able to recognize small molecules in recent years [7,8,9]. The method lies in the polymerization and crosslinking of functional monomers in the presence of a template molecule, which is subsequently extracted from the resulting polymer matrix, generating the cavities complementary in shape and sizes with the template. The material is obtained with the advantage of high affinity and specificity, good physical and chemical stability, ease of preparation, low cost, and possibility of use in harsh environmental conditions. Molecularly-imprinted polymers (MIPs) found potential applications in many fields. The target molecular recognition ability of MIPs can be assessed by the electrochemical, optical or piezoelectric methods [10,11,12,13]. Immunosensors with molecularly-imprinted polymers are bound to be an alternative to traditional immunosensors based on antibodies. This is due to the unique combination of advantages displayed by the artificial materials, including the absence of animal inoculation and sacrifice, unnecessary hapten conjugation to carrier protein for stimulated production, the possibility of manufacturing MIPs against toxic substances, excellent physicochemical stability, reusability, ease of storage, and recognition in organic media [14].

The preparation of MIPs by direct electropolymerization on the sensing surface eliminates the necessity of strict synthesis and film that usually requires preparing spin- or solution-casting technology, offering the advantage of better control of the film thickness at the electrode surface [15]. Integration of nanoparticles in molecularly-imprinting sensors has the benefit of enhancing the catalytic activity of the modified electrode surface [8].

First defined by Yaghi and co-workers in 1995 [16,17], following the pioneering work of Hoskins and Robson, metal-organic frameworks (MOFs) are crystalline porous hybrid materials comprising coupling units (metal ions or metal-oxo units) coordinated by electron-donating organic ligands. MOFs have the highest specific surface areas reported to date for porous materials (up to 10^4^ m^2^ g^−1^) and large internal pore volumes with well-defined pore sizes and apertures. Their principal applications are gas storage, electrode materials for batteries, electrocatalysts for important reactions taking place in fuel cells or electrolyzers, electrode materials for supercapacitors, Li^+^ or H^+^ conductive materials for batteries or fuel cells, and surface films for corrosion inhibition [18,19,20]. Metal organic frameworks (MOF) as highly selective and selective platforms for the development of sensors have attracted growing interests, associated with a fluorimetric detection [21] or with an electrochemical detection [22,23].

**Figure 2:**
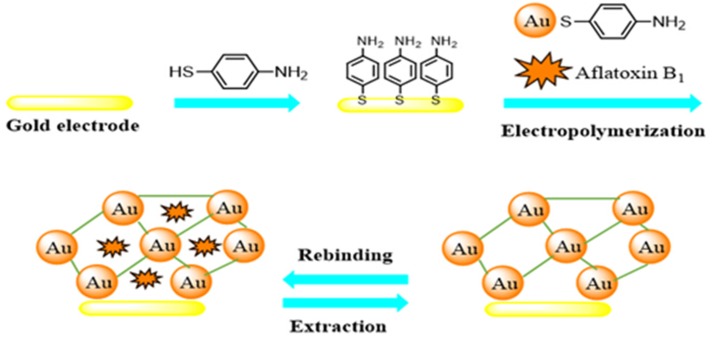
Schematic illustration of the fabrication of the MIP sensor for aflatoxin B1 detection.

Herein, an electrochemical sensor for the sensitive and selective detection of aflatoxin B1, based on a molecularly-imprinted MOF with recognition sites for aflatoxin B1, is prepared through electropolymerization of p-aminothiophenol (PATP)-functionalized AuNPS in the presence of the AFB1 as a template molecule, combining the advantages of molecular imprinting and electrodeposition with those conferred by MOF. The schematic principle of the fabrication of this MIP-MOF sensor for AFB1 detection is presented in Figure 2. To the best of our knowledge this is the first molecularly-imprinted MOF electrochemical sensor reported to detect aflatoxin B1.

## 2. Results and Discussion

### 2.1. Fabrication of Aflatoxin B1 Imprinted MOF Film

The fabrication of the MIP sensor was carried out in several steps as shown in Figure 2.

#### 2.1.1. Characterization of PATP Functionalized AuNPs

**Figure 3:**
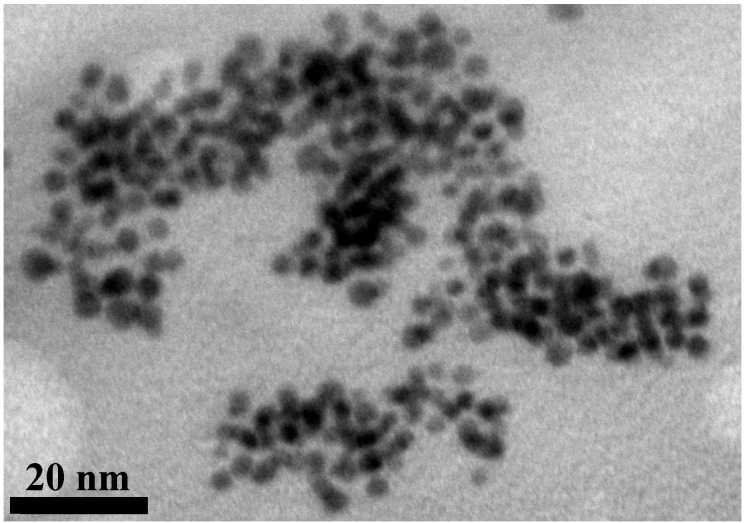
TEM observation of *p*-aminothiophenol-functionalized gold nanoparticles.

TEM observation presented in Figure 3, depicts a mean diameter of around 5 nm with a narrow size distribution.

**Figure 4:**
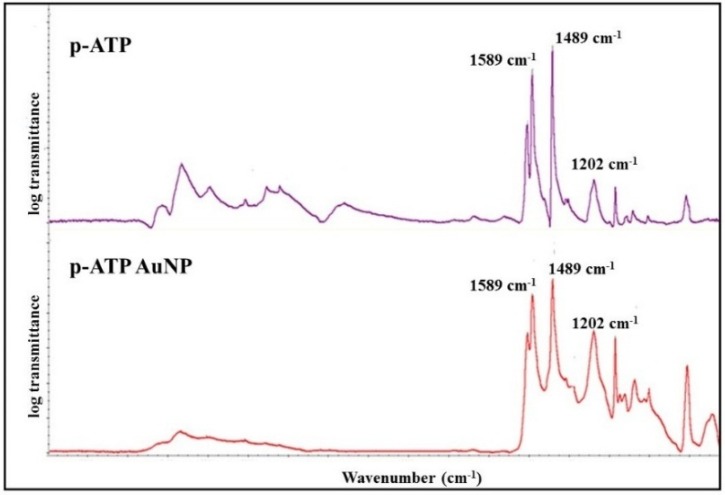
FTIR spectrum of *p*-aminothiophenol (ATP) and of *p*-aminothiophenol-functionalized gold nanoparticles (Au-ATP).

The FTIR spectrum of *p*-aminothiophenol was compared to that of *p*-aminothiophenol-functionalized gold nanoparticles (cf Figure 4). The main peaks of *p*-aminothiophenol are present in both FTIR transmission spectra (1202 cm^−1^, 1489 cm^−1^, 1589 cm^−1^).

#### 2.1.2. Electropolymerization of AFB1 MIP-MOF Film

The first step to prepare the electrodes was the self-assembly of PATP on the gold electrode surface by Au-S bonds and the subsequent deposition of an electropolymerized layer of polythioaniline/AuNPs/aflatoxin. The aflatoxin template was then removed from the matrix, providing cavities that can recognize and bind aflatoxin in the next step.

**Figure 5:**
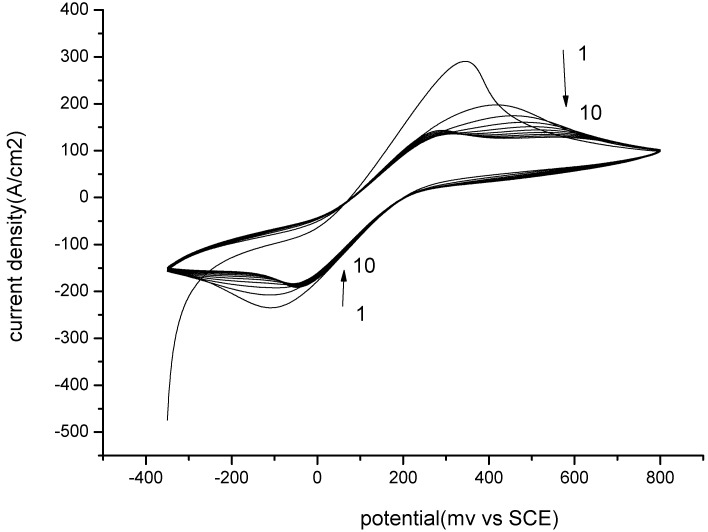
Electropolymerization of MIP by CV. Potential range from −0.35 V to 0.8 V *vs.* SCE. Scan rate 100 mV/s.

The electropolymerizaton was performed by CV in a solution of 10 mM [Fe(CN)_6_]^3−/4−^ in PBS pH 7.2 and the film formation was examined through the changes in the current per cycle. As shown in Figure 5, the current decreased as the number of cycles increased, suggesting gradual growth of a compact polymeric film which covered the surface of the gold electrode, hindering the charge transfer of the redox probe at the electrode. The electrochemical polymerization process consists of intermolecular reactions between the aniline moieties of PATP.

#### 2.1.3. Structural and Electrochemical Characterization of AFB1 MIP-MOF Film

**Figure 6:**
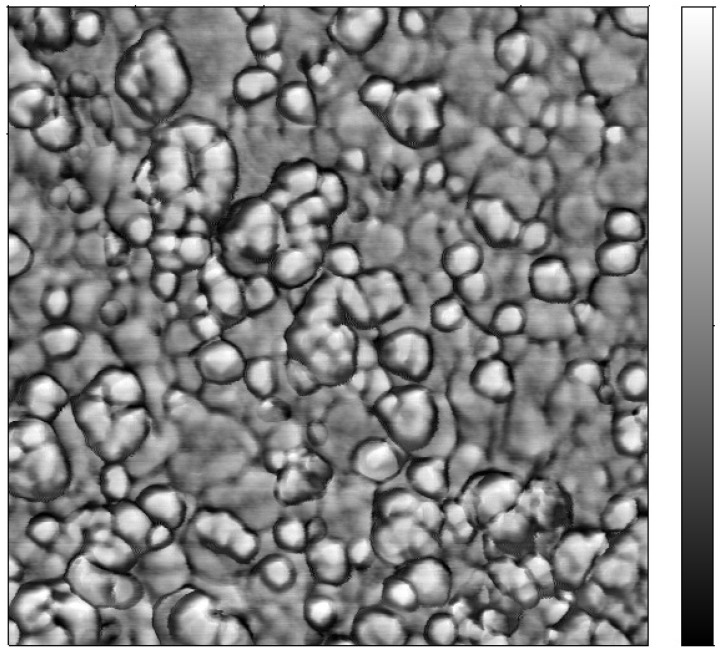
AFM phase image showing gold nanoparticles included in the MIP MOF film. Size of image: 1 µm × 1 µm; Vertical scale: phase from −10.3 degrees to +14.3 degrees.

Figure 6 shows the phase of the prepared film, observed by AFM, which offers important information about the elasticity modulus of the MIP MOF film surface. This hybrid polymer film presents a very high phase variation: 24 degrees. This variation is due to the apparent AuNPs, their apparent diameter being around 30 nm, which is larger than that measured with TEM, due to the curvature radii of the silicon tip used.

**Figure 7:**
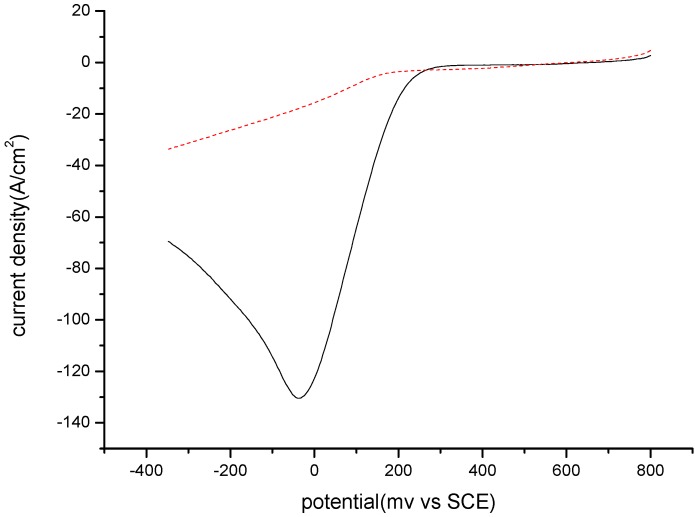
LSV behavior of the MIP (black line) and NIP (dotted red line) sensors after electropolymerization.

After the electropolymerization, MIP and NIP films were characterized using linear sweep voltammetry (LSV), [Fe(CN)_6_]^3−/4−^ being used as a redox mediator. Their electrochemical behavior is quite different, as shown by the voltammograms presented in Figure 7. Charge transfer through NIP film is very low, compared to that through MIP. This shows that their morphology should be quite different and that the presence of a template molecule (AFB1) could help the charge transfer.

### 2.2. Recognition Ability of MIP MOF Films toward Aflatoxin B1

#### 2.2.1. Electrochemical Detection of AFB1 Recognition by MIP-MOF Film

**Figure 8:**
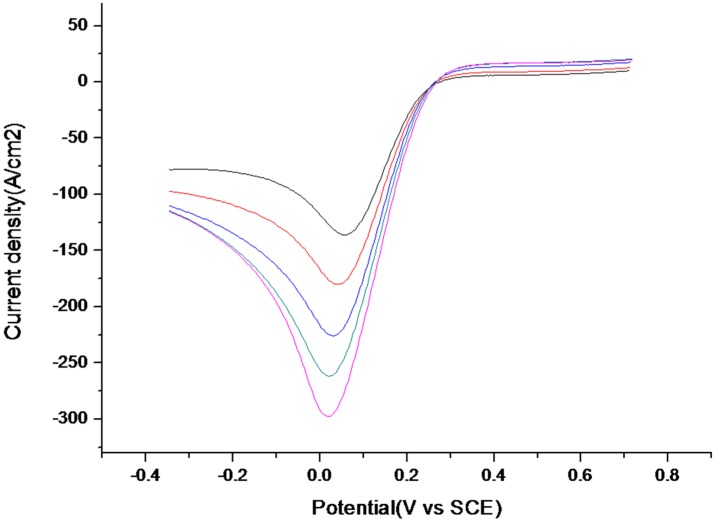
LSV after extraction of the template (black curve) and upon rebinding of various concentrations of aflatoxin: 3.2 × 10^−15^ M (red curve), 3.2 × 10^−12^ M (blue curve), 3.2 × 10^−9^ M (green curve), 3.2 × 10^−6^ M (purple curve). Potential range: 0.8 V to −0.35 V *vs.* SCE. Scan rate 50 mV/s.

In order to check the uptake process for variable concentrations of aflatoxin B1, LSV measurements were performed in a solution of PBS pH 7.2 containing 10 mM [Fe(CN)_6_]^3−/4−^. After the extraction of the template, the prepared MIP was incubated with solution of different concentrations of aflatoxin B1. It was observed that an increase in the current peak and a shift of the peak potentials to a more negative potential with the increase of aflatoxin concentration from 3.2 fM to 3.2 μM (Figure 8) related to the binding of the analyte in the specific cavities. The higher concentration of aflatoxin molecules in MMOF film increases the electron transfer rate.

#### 2.2.2. Mechanism of AFB1 Recognition by MIP-MOF Film

There are π-donor/π-acceptor interaction between aniline moieties and aflatoxin B1 molecules (due to dione functions of AFB1 molecule). The conductivity of the MIP-MOF film is partly based on polythioaniline, a conjugated polymer electron donor. This conductivity is increased when the concentration of AFB1 molecules in the polymer increases. A p-doping of the conjugated polymer is then induced, due to charge delocalization, creating a discrete donor level in the energy band diagram of polythioaniline [24]. This phenomenon can explain how the electron transfer rate of the ferro/ferricyanide redox probe is increased when the concentration of AFB1 increases. This phenomenon has been evidenced through fluorescence quenching [21] and through increase of electron transfer rate for a MIP-MOF film when concentration of nitroaromatic molecules increases [22].

### 2.3. Analytical Performance of the MIP MOF Sensor

**Figure 9:**
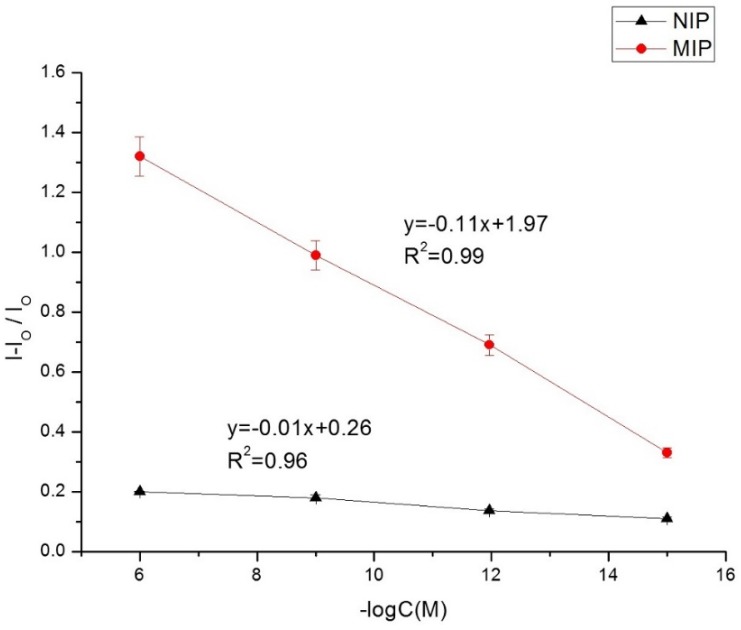
Calibration curves obtained for MIP (red curve) and NIP (brown curve) sensors by LSV for 3.2 fM to 32 nM aflatoxin. Under optimized parameters: extraction time 30 min, incubation time 20 min. *I* = signal after incubation with aflatoxin. *I*_O_ = signal after extraction.

From Figure 9 we can see the dependence of LSV current density on the concentration of aflatoxin. Data points were calculated as the average of three different electrodes. The peak current density ratio (signal-blank)/blank *versus* logarithm function of aflatoxin concentration exhibited a linear response over a range from 3.2 fM to 3.2 μM, with a limit of quantification of 3 fM (1 pg/L) and a limit of detection of 1 fM (0.3 pg/L). The limit of quantification (LOQ) was determined to be 10 σ_b_ and the limit of detection as 3 σ_b_, σ_b_ being the standard deviation of the blank. The low signals obtained for NIP prove the influence of the template in obtaining binding sites for aflatoxin. The imprinting factor (ratio of sensitivity for MIP to sensitivity for NIP) is equal to 11.

As shown in Table 1, compared to previously reported methods (electrochemistry, HPLC/MS), the proposed sensor exhibits better analytical performance in terms of sensitivities.

The fabrication reproducibility was examined with three different modified electrodes, which were constructed independently using the same procedure. LSV studies were performed on each electrode aforementioned for the binding of 3.2 pM of aflatoxin. The precision of the described procedure, in terms of RSD, was 5.6%, which indicated good reproducibility of the fabrication method.

To check the reusability of the proposed sensor, several cycles of extraction and incubation with a solution of 3.2 pM aflatoxin were performed on the same electrode. The sensor maintained its response for three cycles with good reproducibility (RSD 4%). When used in real sample analysis (spiked rice samples), a thorough washing of the sensor surface in a PBS buffer, pH 7.3, is necessary for the complete elimination of the matrix effect.

**Table 1:** Comparison of different methods for the detection of Aflatoxin B1.

Method	LOD	Linear Range	Reference
Immunosensor on screen-printed carbon electrodes	90 pg/mL	0.1–10 ng/mL	[25]
Immunosensor on screen-printed microplate	30 pg/mL	0.05–2 ng/mL	[26]
Immunosensor on glassy carbon electrode	0.07 ng/mL	0.6–2.4 ng/mL	[27]
HPLC	0.25 ng/mL	0.5–10 ng/mL	[28]
HPLC-MSMS	0.08 ng/mL	0.3–10 ng/mL	[29]
LC-MSMS	2.4 pg/mL	0.05–2 ng/mL	[6]
LSV on molecularly imprinted MOF	0.3 fg/mL	1 fg/mL–1 μg/mL	Present method

**Figure 10:**
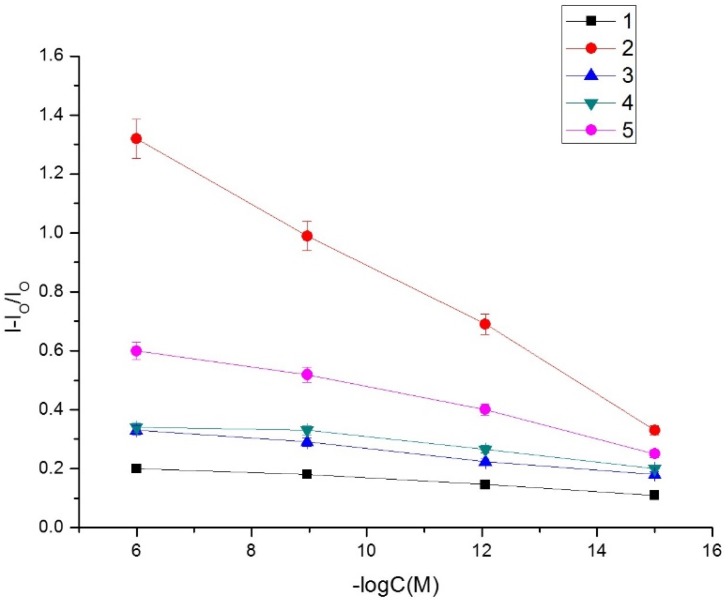
Calibration curves upon analysis of various concentrations of aflatoxins and ochratoxin A. Curve 1: NIP sensor for AFB1; Curve 2: MIP sensor for AFB1; Curve 3: MIP sensor for AFB2; Curve 4: MIP sensor for AFG1; Curve 5: MIP sensor for OTA.

Selectivity is a determining parameter in the analytical features of the MIP-MOF sensor. The responses for mycotoxins of similar structures, such as aflatoxin B2 (AFB2), aflatoxin G1 (AFG1), and ochratoxin A (OTA) have been examined. Figure 10 displays the response for different concentrations of interfering species; the peak current density ratio (signal-blank)/blank *versus* logarithm of different concentrations exhibit a linear response over a range from 3.2 fM to 3.2 μM. The cross-reactivity in percent of the MIP-MOF sensor for AFB2, AFG1, and OTA is reported in Table 2, as one hundred times the ratio of concentration of AFB1 giving a response of MIP-MOF sensor to the concentration of other mycotoxin giving the same response. The results show that the proposed biosensor has high selectivity for aflatoxin B1.

**Table 2:** Cross-reactivity of aflatoxins and ochratoxin *versus* AFB1.

Type of Mycotoxin	Cross-reactivity (%)
Aflatoxin B2	10^−7^
Aflatoxin G1	10^−7^
Ochratoxin A	10^−5^

## 3. Experimental Section

### 3.1. Chemicals and Instrumentation

*p*-Aminothiophenol (PATP), tetrachloroauric acid trihydrate (HAuCl_4_·3H_2_O), potassium ferrocyanide (K_4_[Fe(CN)_6_]), sodium borohydride (NaBH_4_), potassium ferricyanide (K_3_[Fe(CN)_6_]), methanol, and phosphate buffered saline (PBS) tablets were purchased from Sigma-Aldrich (Saint-Quention Fallavier, France). All solutions were prepared using Milli-Q water with a resistivity of 18.2 MΩ·cm at 25 °C. Aflatoxin B1 (1 mg), Aflatoxin B2 (1 mg), Aflatoxin G1 (1 mg), and Ochratoxin A (1 mg) were purchased from Sigma-Aldrich.

UV-VIS absorption spectra of the AuNPs nanoparticles were recorded at room temperature with a Lambda 900 UV/Vis/NIR spectrophotometer (Perkin Elmer, Waltham, MA, USA) between 300 and 800 nm.

Transmission electron microscopy (TEM) pictures were obtained using a CM120 TEM (Philips, Amsterdam, Netherlands) with an accelerating voltage of 120 kV (Centre Technologique des Microstructures-Lyon 1, Villeurbanne, France). AuNPs were examined after deposition of 5 mL of diluted solutions on a formvar-coated copper grid (Electron Microscopy Sciences) and evaporation to dryness.

Infrared spectra were recorded at room temperature using a Continuμm microscope coupled with Nexus infrared spectroscopy in specular reflectance equipped with an MCT detector (Nicolet Instruments Corporation, Madison, WI, USA). Recordings were obtained with a resolution of 4 cm^−1^, a spectral width between 690 and 4000 cm^−1^ and signal processing through Happgenzel apodization. (256 scans).

Electrochemical measurement were performed with a VOLTALAB-80 PGZ/301 potentiostat-galvanostat (Hach, Lange, France), controlled by Voltamaster 4 software. Experiments employed in a three-electrode electrochemical cell, with a gold working electrode (0.07 cm^2^), a platinum plate (0.17 cm^2^) as auxiliary electrode and a saturated calomel electrode(SCE) as reference.

AFM was used to investigate the surface topography of the electropolymerized films and their rheological response (phase). The AFM measurements were carried out using an Agilent 5500 AFM (Agilent Technologies, Palo Alto, CA, USA). Silicon tips with a nominal spring constant of 20 Nm^−1^ were used in tapping mode at a frequency of ~300 kHz.

### 3.2. Preparation of PATP Functionalized AuNPs

The AuNPs were synthesized according to the literature method [30]. A typical preparation of gold nanoparticles is as follows: 31.6 mg (8 × 10^−5^ mol) of tetrachloroauric (III) acid trihydrate was dissolved in 30 mL of methanol in a round 100 mL flask equipped with a condenser. A solution of *p*-aminothiophenol (1.6 × 10^−4^ mol) in 12 mL of a methanol/water (*v/v*) mixture was added dropwise under stirring to the gold salt solution, which changed color from yellow to dark brown. After 10 min, 30.4 mg of NaBH_4_ (8 × 10^−4^ mol) dissolved in 2.2 mL of water was added dropwise to the mixture under vigorous stirring. After 10 min, stop stirring and keep the solution in darkness for 1 h. The suspension was then filtered through a polymer membrane and washed successively with water and ether. The resulting black powder was dried and stored either as a solid or dispersed in 0.1 N HCl solution. The UV-vis spectra of gold nanoparticle colloids show a small surface plasmon band centered around 535 nm. The solid PATP-fuctionalized AuNPs were characterized using TEM and FTIR.

### 3.3. Preparation of MIP and Non-Imprinted (NIP) MOF Modified Electrodes

Both MIP and NIP films were prepared on the surface of gold electrodes using cyclic voltammetry (CV), by electropolymerization of PATP self-assembled on gold electrodes and PATP functionalized gold nanoparticles. Gold substrates were provided by the French RENATECH network (LAAS, CNRS Toulouse, France). They were fabricated using standard silicon technologies. (100)-oriented, P-type (3–5 Ω·cm) silicon wafers were thermally oxidized to grow an 800 nm-thick field oxide. Then, a 30 nm-thick titanium layer followed by a 300 nm thick gold top layer were deposited by evaporation under vacuum. The gold electrodes were 1 × 1 cm^2^ square plates. Prior to SAM formation, the gold electrodes were cleaned by rinsing with ethanol and water followed by exposure to UV/ozone. An intermediate monolayer of PATP was firstly formed by immersing the gold electrodes in a 50 mM PATP ethanol solution for 12 h at 4 °C. After rinsing with ethanol and water to remove the physically adsorbed PATP, the electropolymerizaton of MIP was conducted in a solution containing 0.1 mg/mL PATP functionalized AuNPs and 0.1 mg/mL aflatoxin B1 in the supporting electrolyte (10 mM Fe(CN)_6_^3−/4−^ in PBS pH 7.2). The potential was cycled between −0.3 V and +0.80 V *vs.* SCE, at a scan rate of 100 mv/s, for 10 cycles. A control non-imprinted film (NIP) was also prepared in every case, with the same method, but without any additional aflatoxin B1.

The modified electrodes were then washed with a 1:4 (*v/v*) mixture of methanol/PBS solution for 30 min to remove the imprinted molecules and adsorbed molecules on the surface of the film, triggering the formation of recognition cavities in the MIP film.

In order to promote the recognition and binding of AFB1 molecules, the MIP electrode was left to incubate with aflatoxin B1 solutions for 20 min. For the characterization of the MIP sensor at different steps of the preparation, electrochemical measurements were carried out in 10 mM [Fe(CN)_6_]^3−/4−^ in PBS pH 7.2 at room temperature. Linear sweep voltammetry was employed from 0.80 V to −0.35 V at a scan rate of 50 mV/S.

## 4. Conclusions

The development of an electrochemical sensor for sensitive detection of aflatoxin B1 based on an MIP-based polymer/AuNPs hybrid membrane is reported for the first time herein. The MIP was prepared on the surface of PATP-functionalized gold electrodes by electropolymerization of PATP-functionalized AuNPs in the presence of AFB1 as the template molecule. The developed sensor showed a wide linear range between 3.2 fM and 3.2 µM with an LOQ of 3 fM, lower than that reported for other AFB1 sensors. With good sensitivity and reproducibility, the MIP hybrid sensor was shown to have the potential to be an effective method for the selective electrochemical detection of AFB1 in food samples. The easy fabrication and robustness of molecularly-imprinted MOF films make them ideal candidates for the development of sensing devices.
